# NF-κB in neurodegenerative diseases: Recent evidence from human genetics

**DOI:** 10.3389/fnmol.2022.954541

**Published:** 2022-08-02

**Authors:** Barbara Kaltschmidt, Laureen P. Helweg, Johannes F. W. Greiner, Christian Kaltschmidt

**Affiliations:** ^1^Department of Molecular Neurobiology, Bielefeld University, Bielefeld, Germany; ^2^Forschungsverbund BioMedizin Bielefeld, Ostwestfalen-Lippe (OWL) (FBMB E.V.), Bielefeld, Germany; ^3^Department of Cell Biology, Biological Faculty, University of Bielefeld, Bielefeld, Germany

**Keywords:** NF-κB–nuclear factor kappa B, nervous system, Alzheimer’s disease, Parkinson’s disease, glioblastoma multiforme (GBM), SHARPIN, PARKIN, HOTAIR

## Abstract

The transcription factor NF-κB is commonly known to drive inflammation and cancer progression, but is also a crucial regulator of a broad range of cellular processes within the mammalian nervous system. In the present review, we provide an overview on the role of NF-κB in the nervous system particularly including its constitutive activity within cortical and hippocampal regions, neuroprotection as well as learning and memory. Our discussion further emphasizes the increasing role of human genetics in neurodegenerative disorders, namely, germline mutations leading to defects in NF-κB-signaling. In particular, we propose that loss of function mutations upstream of NF-κB such as ADAM17, SHARPIN, HOIL, or OTULIN affect NF-κB-activity in Alzheimer’s disease (AD) patients, in turn driving anatomical defects such as shrinkage of entorhinal cortex and the limbic system in early AD. Similarly, E3 type ubiquitin ligase PARKIN is positively involved in NF-κB signaling. PARKIN loss of function mutations are most frequently observed in Parkinson’s disease patients. In contrast to AD, relying on germline mutations of week alleles and a disease development over decades, somatic mutations affecting NF-κB activation are commonly observed in cells derived from glioblastoma multiforme (GBM), the most common malignant primary brain tumor. Here, our present review particularly sheds light on the mutual exclusion of either the deletion of NFKBIA or amplification of epidermal growth factor receptor (EGFR) in GBM, both resulting in constitutive NF-κB-activity driving tumorigenesis. We also discuss emerging roles of long non-coding RNAs such as HOTAIR in suppressing phosphorylation of IκBα in the context of GBM. In summary, the recent progress in the genetic analysis of patients, particularly those suffering from AD, harbors the potential to open up new vistas for research and therapy based on TNFα/NF-κB pathway and neuroprotection.

## Introduction: What is NF-κB?

**FIGURE 1:**
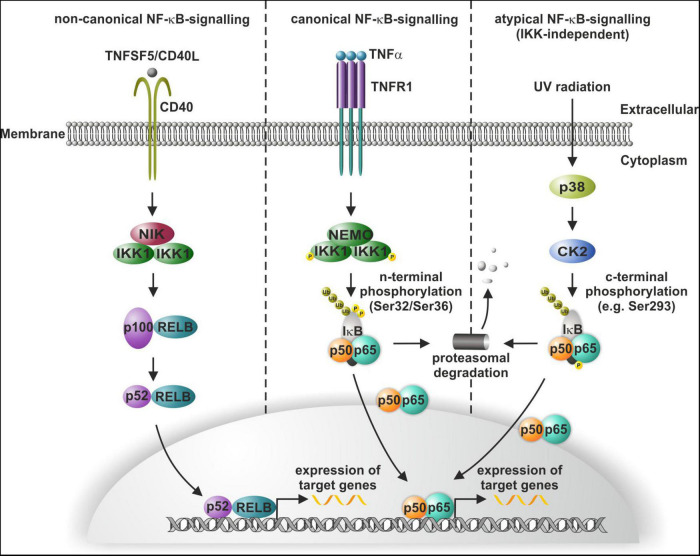
Non-canonical, canonical, and atypical NF-κB-signaling. Non-canonical signaling includes CD40-mediated activation of NF-κB-inducible kinase (NIK), which activates (IκBα) kinase (IKK) and leads to the processing of the p100/RELB and translocation of p52/RELB **(left panel)**. In canonical NF-κB-signaling, TNFα-mediated activation of the IKK complex leads to phosphorylation of IκBα and allows the p50/p65 dimer to translocate to the nucleus **(mid panel)**. UV light induces atypical activation of NF-κB-signaling through p38-MAPK activation and consequent IκBα phosphorylation independent to IKK, which again releases the p50/p65 dimer **(right panel)**. Modified from Kaltschmidt et al. (2021).

The transcription factor nuclear factor kappa light chain enhancer of activated B cells (NF-κB) is a key regulator of inflammation and cancer progression and vitally drives a broad range of cellular processes within the mammalian nervous system (Kaltschmidt and Kaltschmidt, 2009). In the present review, we will particularly shed light on this crucial role of NF-κB in the nervous system as well as in neuroprotection and associated diseases like Alzheimer’s disease (AD), Parkinson’s disease (PD), and glioblastoma multiforme (GBM). On the molecular level, the NF-κB family comprises five DNA-binding members REL (c-REL), RELA (p65), and RELB (RELB) as well as NFKB1 (p50) and NFKB2 (p52), with the latest lacking transactivation domains (Ghosh et al., 1995). NF-κB-signaling can be distinguished into the canonical, non-canonical and atypical pathway (Figure 1). Canonical and non-canonical NF-κB-signaling share a common regulatory element, the inhibitor of κB (IκBα) kinase (IKK). In non-canonical NF-κB-signaling, ligand binding to receptors like CD40 activates IKK1 *via* NF-κB-inducible kinase (NIK). Phosphorylation of IKK1 results in processing of p100 to p52 and subsequent nuclear translocation of p52/RELB (Kaltschmidt et al., 2018; Figure 1). Canonical NF-κB-signaling is mediated by phosphorylation of the NEMO/IKK1/IKK2-complex for instance upon binding of ligands to TNF receptor 1 (TNFR1) (Figure 1). Phosphorylated IKKs in turn phosphorylate IκBα, which keeps p65/p50 in an inactive cytoplasmic state (Zhang et al., 2017). IKK-dependent phosphorylation of IκBα results in its proteasomal degradation and enables nuclear translocation of p65/p50 and expression of target genes (Figure 1). In atypical NF-κB-signaling, exposure of cells to UV light leads to NF-κB activation *via* p38 MAP Kinase (MAPK) activation, in turn resulting in casein kinase 2 (CK2)-mediated phosphorylation and degradation of IκB. In contrast to canonical and non-canonical activation of NF-κB, CK2-dependent atypical phosphorylation of IκB is c-terminal (e.g., at Ser293) and independent to IKK, which phosphorylates IκB at n-terminal phosphorylation sites (such as Ser32 or Ser36) (McElhinny et al., 1996; Sayed et al., 2000; Kato et al., 2003; reviewed in Lin et al., 2010; Figure 1). Next to TNFα (Furukawa and Mattson, 1998; Kaltschmidt et al., 1999; Figure 1), NF-κB can be also activated by the neurotransmitter glutamate (Guerrini et al., 1995; Kaltschmidt et al., 1995) and its agonists like kainate (Kaltschmidt et al., 1995) and N-methyl-D-aspartate (NMDA) (Meffert et al., 2003) in the nervous system as discussed below.

## Overview on the role of NF-κB in the nervous system

When we started in 1992 to work on NF-κB in the nervous system not much was known on that topic. Our own review (Kaltschmidt B. et al., 1993) on this topic suggested an involvement of NF-κB based on the production of reactive oxygen intermediates in various neurological diseases, such as multiple sclerosis (MS), Alzheimer’s disease (AD), Parkinson’s disease (PD), and others. Today, a Pubmed search with MeSH entries “nervous system disease” and “NF-kappa B” reveals 4,750 results (May 18, 2022), starting in 1993 with two of our own papers.

## Synaptosomes

Our starting investigation was a biochemical study with fractionation of rodent brains in so called synaptosomes. Synaptosomes are prepared by shear stress with a Potter type tissue homogenizer followed by gradient fractionation from different brain regions (Whittaker et al., 1964). Why we used synaptosomes? This biochemical preparation provides the opportunity to fractionate the complex brain in simple subcellular compartments. In addition, synaptosomes are a way to enrich NF-κB from total brain lysates. Synaptosomes can be analyzed by standard DNA-binding techniques such as EMSA to identify transcription factors such as NF-κB.

Synaptosomes are composed of pre-synaptic elements stabilized by post-synaptic density elements. Within synapses derived from olfactory bulb, white and gray matter, cerebellum and brain stem we could detect inducible NF-κB activity, with highest activity in gray matter. DNA binding of NF-κB could be inhibited by recombinant IκBα (Kaltschmidt C. et al., 1993), previously called MAD-3. Later on, synaptic localization of NF-κB could be reproduced by several other groups using synaptosomes (e.g., Meberg et al., 1996; Suzuki et al., 1997; Meffert et al., 2003; Schmeisser et al., 2012). In this line, it was reported that RELA could exist in two pools, a soluble one in synaptosomes and a membrane bound in fractions containing post-synaptic density proteins. While the active RELA-pool in the synaptoplasm is initially activated during early memory consolidation, its subsequent translocation into the membranes of the synaptosomes seems important for consolidation of long-term memory in mice (Salles et al., 2015). Furthermore, localization in post-synaptic spines and regulation of synapse growth was shown by Meffert et al. (2003) (Boersma et al., 2011; Dresselhaus et al., 2018). The function of transcription factors in synaptosomes could be within “a dialogue between synapses and genes” as published by Kandel (2001). In this hypothesis a feedback of activated synapses back to the nucleus (so called retrograde transport, see below) can initiate changes in gene transcription. These might direct synaptic enhancement and regrowing of novel synaptic contacts. For this task inducible transcription factors are well suited. Collectively these gathered evidence suggest that NF-κB is a synaptic protein in different brain region such as cerebellum, cortex and hippocampus, olfactory bulb, and brain stem.

## Retrograde transport in neurons

We could show that NF-κB could be retrogradely transported from the pre-synaptic site to the nucleus in hippocampal neurons (Wellmann et al., 2001). Retrograde transport could be studied by injection of radioactively labeled proteins in animals, like it was shown for transport of nerve growth factor (NGF) in sensory neurons (Stoeckel et al., 1975). Furthermore transport of green fluorescent protein (GFP) fusion proteins could be analyzed by life microscopy (Wellmann et al., 2001), which could be combined with the analysis of transport in photobleached regions (Meffert et al., 2003). Finally transport of single fusion proteins could be studied with blinking probes (Widera et al., 2016). Later on, we could extend this observation with super resolution microscopy (Mikenberg et al., 2007; Widera et al., 2016). In this line, it is important to introduce TNFα, which also can be retrogradely transported in inflammatory lesions of peripheral nerves (Shubayev and Myers, 2001). Accordingly, we demonstrated Hsc70 as a novel interactor of NF-κB potentially involved in retrograde transport in rodent brain (Klenke et al., 2013), while deoxyspergualin, a specific inhibitor of Hsc70, inhibited nuclear import of NF-κB. We have previously shown that NF-κB was activated in microglia of experimental allergic encephalomyelitis (EAE) rats (Kaltschmidt et al., 1994a). In this line, effective treatment of EAE with deoxyspergualin could be shown in two models of acute and chronic relapsing EAE (Schorlemmer and Seiler, 1991). Taken together these data provide evidence for a retrograde transport of NF-κB from activated synapses to the nucleus, one component of the transport complex is Hsc70, which could be inhibited by deoxyspergualin.

## Constitutive NF-κB activity in neurons

We discovered constitutive activity (always activated NF-κB in the nucleus) within cortical and hippocampal regions of rodent brains (Kaltschmidt et al., 1994b). Further analysis of NF-κB activity with reporter gene assays could reproduce this constitutive activity within the neurons of the cortical or the hippocampal region (Schmidt-Ullrich et al., 1996; Bhakar et al., 2002). In particular, Barker and coworkers showed that efficient blocking of endogenous NF-κB activity by recombinant adenovirus led to death of cortical neurons, whereas induction of NF-κB activity resulted in neuroprotection (Bhakar et al., 2002). Evidence for constitutive activity demonstrates that NF-κB is a sensor for neuronal activity of glutamatergic neurons. Anesthesia of cultured neurons with, e.g., NMDA receptor antagonists inhibits constitutive NF-κB activity.

## Inducible NF-κB in neurons

Activation of NF-κB in neurons is possible by the neurotransmitter glutamate (Guerrini et al., 1995; Kaltschmidt et al., 1995) and agonists such as kainate (Kaltschmidt et al., 1995) and NMDA (Meffert et al., 2003), as well as by pro-inflammatory cytokines as TNFα (Furukawa and Mattson, 1998; Kaltschmidt et al., 1999; Figure 1). Taken together evidence indicated a major role of NF-κB in glutamatergic signaling as well as pro-inflammatory TNF mediated signaling.

## Animal models for studying NF-κB in the nervous system

In the beginning of the 21st century, several new transgenic mouse models with specific repression or activation of NF-κB in the nervous system were generated, allowing studies of behavior and learning. Mollie Meffert and coworkers discovered that p65-deficient mice have no synaptic NF-κB. These mice when crossed to TNFR1-deficient mice could survive from TNF-mediated hepatic failure and had a learning defect of spatial learning in a radial arm maze (Meffert et al., 2003). We discovered that expression of transdominant negative IκBα in glutamatergic neurons affected spatial memory formation and repressed expression of the novel NF-κB target gene protein kinase A (catalytic subunit α) (Kaltschmidt et al., 2006). Warner Greene and coworkers discovered that neuronal expression of super-repressor IκB in GABA-ergic interneurons led to enhanced spatial learning and memory (O’Mahony et al., 2006). Later on, it was shown that NF-κB-repression by transdominant negative IKK2 led to impaired learning and memory. The authors further identified insulin-like growth factor 2 (IGF2) as a novel IKK/NF-κB target gene. In this line, IGF2 was able to restore synapse density and promoted spine maturation in IKK/NF-κB signaling-deficient neurons within 24 h (Schmeisser et al., 2012). In contrast, expression of a constitutively active allele of IKK2 in forebrain neurons led to degeneration of microglia and astrocytes as well as spatial learning defects (Maqbool et al., 2013). Therefore evidence indicates NF-κB involvement in learning and memory and additionally in brain regeneration *in vivo*.

## Germline mutations affecting NF-κB activation in the context of Alzheimer’s disease

**FIGURE 2:**
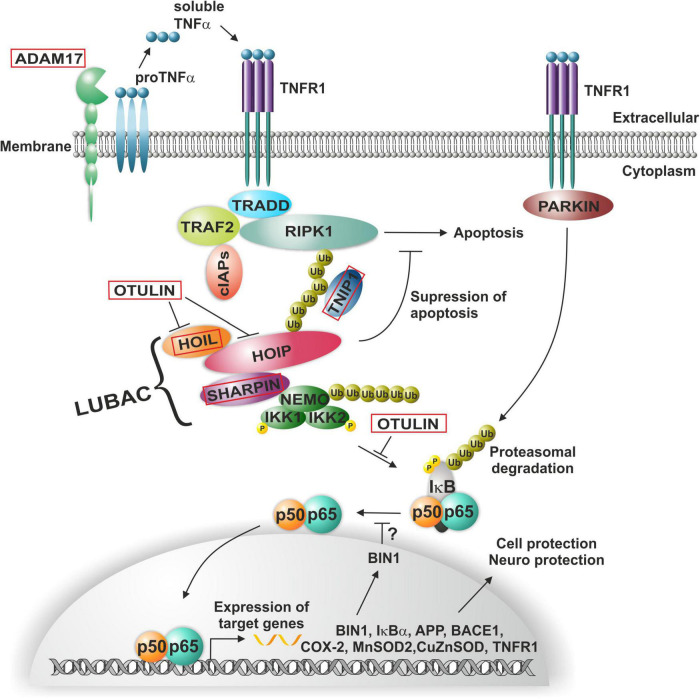
NF-κB-signaling in Alzheimer’s and Parkinson’s disease. ADAM17-induced soluble TNFα mediates the activation of the TNF receptor complex, where poly-ubiquitination leads to the recruitment of the LUBAC complex. Linear ubiquitination of NEMO mediates the activation and phosphorylation of the (IκBα) kinase (IKK) complex and consequent activation of NF-κB p50/p65, in turn driving expression of a range of AD-relevant genes such as BIN1, IκBα, APP, BASE1, COX-2, MnSOD, CuZnSOD, or TNFR1. In PD, activation of NF-κB is mediated by PARKIN in a TNFR1-dependent manner.

Alzheimer’s disease (AD) is a devastating neurodegenerative disease and was discovered by Alois Alzheimer in Munich when analyzing brain sections of his patient Auguste Deter, who died in a lunatic asylum in Frankfurt. Alois Alzheimer immediately discovered the presence of “miliary foci” (senile plaques) and very strange changes in neurofibrils, which clump into tangles that eventually replace dead neurons (Alzheimer, 1907). Later on, he extended this observation toward examples of neurofibril tangles and plaques (Alzheimer, 1911). Then, Konrad Beyreuther and coworkers purified plaque proteins and determined the amyloid plaque core protein sequence (Masters et al., 1985). Furthermore, the amyloid A4 beta precursor protein was cloned by the group of Müller-Hill (Kang et al., 1987). Notably, many AD-relevant genes such as BIN1, IκBα, APP, BASE1, COX-2, MnSOD, CuZnSOD, TNFR1, and others are target genes of NF-κB (Snow and Albensi, 2016; Figure 2).

**FIGURE 3:**
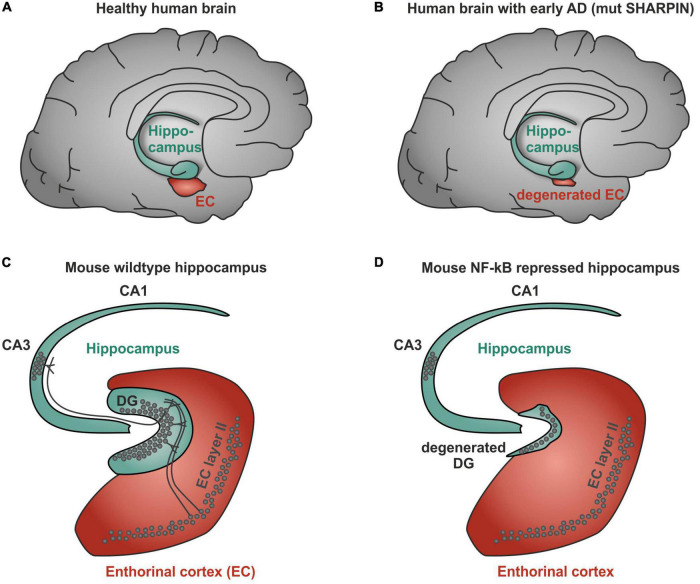
NF-κB-mediated degeneration of human and mouse brain. **(A)** Scheme of healthy human brain showing a normal sized entorhinal cortex. **(B)** Human SHARPIN mutated Alzheimer’s disease (AD) brain showing a degenerated entorhinal cortex. **(C)** Scheme of healthy NF-κB wildtype mouse brain with normal dentate gyrus. **(D)** NF-κB repression in mice brains leads to a degenerated dentate gyrus.

However, a recent genetic study using genome wide association of 111,326 clinically diagnosed AD cases and 677,663 controls challenges this one and only hypothesis of the A beta concept and provides additional evidence for AD-linked genes with higher significance. Notably, BIN1 (bridging integrator 1) alleles (*p*-value: 6 × 10^−118) were most significantly associated to AD, whereas APP (precursors of A beta) had a significance of correlation of only 1 × 10^−12 (Bellenguez et al., 2022). Most interestingly, BIN1 knockout in breast cancers was linked to increased nuclear NF-κB (Ghaneie et al., 2006), suggesting a dual role as inhibitor of NF-κB and as a target gene (Mao et al., 1999; Figure 2). BIN1 encodes several isoforms of a nucleocytoplasmic adaptor protein, initially described with tumor suppressor functions. BIN1 may be involved in the regulation of MYC activity and the control of cell proliferation (Sakamuro et al., 1996). Three isoforms of BIN1 were detected in neurons and astrocytes (isoforms 1, 2, and 3) and four isoforms in microglia (isoforms 6, 9, 10, and 12) of human brain (Taga et al., 2020) showing a strong association of BIN1 isoforms expressed by neurons/astrocytes and tangles that might contribute to cognitive decline in AD. Its role as a tumor suppressor gene suggests a function of wt BIN1 allele as an inhibitor of NF-κB activation (Elliott et al., 2000). Additionally, BIN1 transcript levels were increased in AD brains and BIN1 mediates AD risk by modulating Tau pathology (Chapuis et al., 2013). In particular, Chapuis et al. (2013) identified a novel 3 bp insertion allele upstream of BIN1, which is directly linked to increased BIN1 expression and risk for developing AD. In addition, this novel insertion was associated with Tau accumulation but not with amyloid loads in AD brains. On mechanistic level, knockdown of the BIN1 ortholog Amph suppressed Tau-toxicity in an amyloid-independent manner in *Drosophila*. Coimmunoprecipitation studies further substantiated a direct interaction between Tau and BIN1 in human cells and mouse brains. Although the particular pathogenic mechanism linking BIN1 and Tau in AD progression remains unclear, BIN1 may modulate Tau aggregation/oligomer formation in earlier disease stages (Chapuis et al., 2013). In human induced neurons, BIN1-knockout reduced early endosome signaling and overexpression of the AD-relevant isoform of BIN1 (isoform 1) increased the size of early endosomes and led to neurodegeneration (Lambert et al., 2022). The TNFα pathway with linear ubiquitination was also included in the new genetically associated processes within AD patients (Bellenguez et al., 2022). Interestingly, ADAM17, an extracellular protease leading to soluble TNFα, the TNF receptor interactor TNIP1, and components of the linear ubiquitination machinery linear ubiquitination chain assembly complex (LUBAC): HOIL, SHARPIN, and the negative regulator OTULIN are all affected by mutations in AD patients (Figure 2, red boxes). In this context, it might be interesting to note that Mattson and coworkers discovered a neuroprotective effect of TNFα (Cheng et al., 1994). Furthermore, they discovered that TNFα protected against A beta/glutamate toxicity (Barger et al., 1995; Albensi and Mattson, 2000). Activated NF-κB might direct the expression of antioxidant enzymes such as MnSOD and Calcium-binding proteins such as calbindin. Calcium buffering and reduction of reactive oxygen intermediates by antioxidant enzymes can actively protect against glutamate and Ab mediated induction of apoptosis (see for further discussion: Neuroprotective signal transduction edt. M.P. Mattson Springer Science Press 1998). We could reproduce this observation in cerebellar granule cells and additionally could show that treatment with a low dose of A beta (100 nM) activates p65 specifically and fortifies neurons against a neurotoxic high dose of A beta (Kaltschmidt et al., 1999). In addition also TNFα activated NF-κB at a very sharp peak of 2 ng/ml leading to neuroprotection whereas higher doses of TNF had adversive effects. Furthermore, we could demonstrate that activated NF-κB was restricted to cells in the close vicinity of early plaques in AD patient brains (Kaltschmidt et al., 1997; Ferrer et al., 1998). Interestingly, the overall NF-κB immunoreactivity was quite low in AD brains in comparison to age-matched controls. Recent genetic evidence of potential loss-of-function mutants involved in NF-κB signaling of AD patients might provide a genetic reason for this early correlation (Bellenguez et al., 2022). In this line, human SHARPIN mutants were shown to have less capability of NF-κB activation (Park et al., 2021). Furthermore, SHARPIN mutants (Park et al., 2021) of the LUBAC complex are highly significantly correlated with a reduction in the thickness of the entorhinal cortex, one of the earliest signs of AD (Kobro-Flatmoen et al., 2021; Figures 3A,B). Soheili-Nezhad et al. (2020) identified a SHARPIN mutant in AD associated with degeneration of the limbic system and its interconnecting white-matter. Since SHARPIN is a post-synaptic density protein and other components of the TNFα pathway (see also Figure 2) are also localized in neurons, we conclude that some mutations present in AD led to a reduced TNFα/NF-κB-driven neuroprotection. Accordingly, a transgenic mouse model with NF-κB ablation in basal forebrain neurons led to a severe degeneration of the dentate gyrus (Figures 3C,D), which could be rescued on the cellular level by reactivation of NF-κB (Imielski et al., 2012). In addition, behavioral deficits were completely repaired. In summary, our discussion emphasizes a potential neuroprotective, regenerative role of NF-κB in neurons of AD. Based on the evidences reported above, we suggest a testable hypothesis where loss of function mutations upstream of NF-κB such as ADAM17, SHARPIN, HOIL, or OTULIN (Figure 2) lead to defects in NF-κB signaling including retrograde transport of the signaling endosome and neuroprotection. Finally, these defects might account for the anatomical defects seen in the entorhinal cortex (early AD, Figure 3) followed up by the limbic system and finally shrinkage of the cortex (late AD). We hypothesize based on the evidence presented above that the development of the disease is observed over decades of continuous cognitive decline because the defects in AD patients all rely on weak alleles in contrast to knockouts in somatic cells as in GBM (see below). In summary these genetic data demonstrate the involvement of TNF mediated NF-κB activation in neurons of AD patients. Especially loss of function mutations of the NF-κB activator Sharpin alone or together with additional mutations suggest a loss of neuroprotection enhancing the development of AD.

## Germline mutations affecting NF-κB activation in the context of Parkinson’s disease

Parkinson’s disease is another important neurodegenerative disease affecting more than approximately 1% of individuals older than 60 years. It is suggested that oxidative stress induces apoptosis of dopaminergic neurons in the substantia nigra, resulting in neurodegenerative disorders observed in patients diagnosed with PD. PD was first described by James Parkinson in 1817 (Parkinson, 2002), as a disease separated from other neurological disorders and characterized by typical involuntary tremulous motions of body parts, even when not in action. Much later Arvid Carlson discovered the presence of dopamine in the brain (Carlsson et al., 1958). He used a fluorimetric method in combination with ion exchange chromatography. Furthermore he discovered that reserpine isolated from the plant Rauwolfia led to a Parkinson like phenotype in rabbits. Reserpine lead to complete depletion of dopamine which could be restored by injection of L-Dopa. This discovery was rewarded in 2000 with the Nobel Price. The biochemical discovery of replenishing dopamine by L-Dopa was translated to a clinical L-Dopa therapy for PD patients (Ehringer and Hornykiewicz, 1960). Neuropathological analysis revealed neuronal lesions in regions of the motor system rich in dopamine such as the substantia nigra but also within the limbic system and the brain stem. The cellular basis of dopamine release are dopaminergic neurons which release dopamine to other neurons without any direct synaptic contacts (volume transmission) and are relatively few (about 4,00,000) in the human brain (reviewed in Schultz, 2007). Lewy (1912) discovered a hallmark of PD pathology the so called Lewy bodies as neuronal eosinophilic cytoplasmic inclusion bodies in brain stems of PD patients. Neurons dying during the progression of PD are characterized by the presence of Lewy bodies in their perikarya and Lewy neurites in their neuronal processes. Lewy bodies can be immunostained with antibodies raised against α-synuclein. This type of immunostaining reveals many kinds of Lewy bodies ranging from dot- or thread-like forms to very large types (Braak and Braak, 2000). Similar to AD in PD changes in the neuronal cytoskeleton develop but in only a few susceptible types of nerve cells. α-synuclein is a small protein involved in synaptic dopamine release and a major component which is aggregated in a prion-like fashion in Lewy bodies (Steiner et al., 2018). Braak et al. (2003a) developed a new staging system for PD severity using anti- alpha synuclein histochemistry. Based on these data, they proposed a new hypothesis on the pathology of PD: alpha synuclein pathology typical for PD might spread from gut to brain presumable along the vagus nerve (Braak et al., 2003b). Later on indeed gut brain transmission of pathology with concomitant loss of dopaminergic neurons could be observed in mice after injection of prion-like alpha synuclein fibrils into gut muscles (Kim et al., 2019). While exogenous alpha synuclein could lead to mitochondrial disfunction, overexpression of parkin could rescue the mitochondrial disfunction (Wilkaniec et al., 2021).

A long established genetic linkage is the loss of function of Parkin E3 ubiquitin ligase (Panicker et al., 2021). In this line, recent *in vitro* analysis showed that overexpression of PARKIN strongly enhances TNFα-mediated NF-κB activation in HEK-293 cells, suggesting an involvement of this E3 ubiquitin ligase in TNFα-mediated NF-κB signaling by stabilizing LUBAC (Meschede et al., 2020; Figure 2).

Furthermore reactive oxygen-mediated neurodegeneration was shown to regulate mRNA expression and survival in neurons derived from neural crest derived stem cells in a sex-specific manner (Ruiz-Perera et al., 2018). In particular we observed that female neurons are more susceptible to oxidative stress-mediated cell death. However, activation of NF-κB (RELA) by TNFα let to significant neuroprotection against oxidative stress-induced cell death in both sexes. While female neurons upregulated the NF-κB target genes SOD2 and IGF2 to induce neuroprotection, male neurons showed elevated expression of the neuroprotective NF-κB target gene PKA cat alpha (Ruiz-Perera et al., 2018). But when cRel, a crucial regulator of neuronal development from neural crest is inhibited, neural crest derived stem cells shift their fate from the neuronal to the oligodendrocytic lineage (Ruiz-Perera et al., 2020; reviewed in Greiner et al., 2019). We therefore conclude that NF-κB seems to be a key player in neuroprotection of both sexes, although the protective gene expression program beneath is sexually dimorphic.

## Somatic mutations affecting NF-κB activation in the context of glioblastoma multiforme

Glioblastoma multiforme (GBM) is the most common malignant primary brain and CNS tumor with a very poor prognosis. GBM rapidly developing *de novo* are classified as primary GBM, while GBM developing from low-grade astrocytoma are classified as secondary GBM and show IDH1 mutations (Ohgaki and Kleihues, 2013). NF-κB signaling has been shown to play a central role in glioblastoma growth (reviewed in Smith et al., 2008). Consistently, mesenchymal differentiation mediated by NF-κB has been shown to promote radiation resistance in GBM (Bhat et al., 2013) and inhibition of NF-κB attenuates mesenchymal characteristics and cell proliferation (Wang et al., 2018). Several studies revealed constitutive activation of NF-κB in GBM cells, which promoted invasiveness, angiogenesis and stem cell features such as self-renewal (Raychaudhuri et al., 2007; Xie et al., 2010; Rinkenbaugh et al., 2016; reviewed in Kaltschmidt et al., 2022). Accordingly, we previously reported the GO term “NF-κB binding” to be enriched in a global transcriptome analysis of three primary glioblastoma stem cell populations (Witte et al., 2021).

**FIGURE 4:**
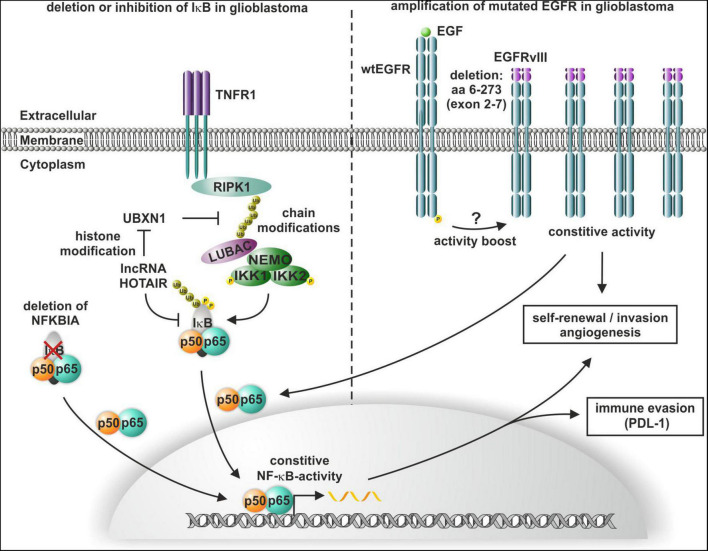
Constitutive activation of NF-κB in glioblastoma multiforme (GBM). Heterozygous deletion of NFKBIA or HOTAIR-mediated phosphorylation of IκB leads to the constitutive activation of NF-κB, which in turn induces self-renewal, angiogenesis, and immune escape. NF-κB is also activated by constitutive activity of mutated EGFRvIII, which also confers self-renewal and invasion in GBM.

The oncogenic long non-coding RNA (lncRNA) HOTAIR induces altered histone H3 lysine 27 methylation and thus has been shown to promote the enrichment of the TNFα/NF-κB signaling protein complex, the IκB kinase complex, and the IKK1-IKK2 complex in glioma cells (Wang et al., 2021). Additionally, HOTAIR suppresses the expression of the NF-κB upstream inhibitor UBXN1 *via* histone modification, leading to enhanced IκBα phosphorylation and consequent NF-κB activation, which in turn induces the expression of PD-L1 and enhances T cell killing resistance and immune escape (Chernorudskiy and Gainullin, 2013; Wang et al., 2021; Figure 4). In addition to enhanced phosphorylation, a heterozygous deletion of NFKBIA (IκBα) has been reported by Bredel and coworkers in 28% of GBM and 22% of cancer stem-like cells. Analyzing 790 human GBMs revealed a mutual exclusion of either NFKBIA deletion or EGFR amplification with similar clinical outcomes (Bredel et al., 2011; Figure 4). Of note, viral-mediated expression of NFKBIA in established GBM cell lines with NFKBIA deletions or EGFR amplification reduced cell viability, but it did not affect NFKBIA/EGFR wildtype GBM cells. EGFR mutations and consequent overexpression are frequent in GBM, with a majority of GBM containing the constitutively active mutant EGFR, which shows a genetic deletion of exon 2–7 and is named EGFRvIII (Heimberger et al., 2005; Figure 4). Other EGFR mutations in GBM include a N-terminal deletion (EGFRvI), deletion of exons 14–15 (EGFRvII), deletion of exons 25–27 (EGFRvIV), and deletion of exons 25–28 (EGFRvV) (An et al., 2018). EGFRvIII overexpression in GBM patients with EGFR amplification was significantly correlated to poor overall survival (Shinojima et al., 2003). On cellular level, several studies linked expression of EGFRvIII in GBM with tumor invasion as well as angiogenesis and inhibition of EGFRvIII inhibited these processes (Zheng et al., 2014; Camorani et al., 2015; Eskilsson et al., 2016; reviewed in Keller and Schmidt, 2017; Figure 4). In EGFRvIII overexpressing glioma cells, angiogenesis and tumor growth were promoted by EGFRvIII-mediated activation of NF-κB (Bonavia et al., 2012). EGFRvIII is also linked to self-renewal of GBM cells and connected to GBM stem-like cell populations (Emlet et al., 2014; Kim et al., 2021). Consistently, EGFRvIII and EGFR are downregulated upon differentiation of GBM neurospheres as well as inhibited EGFR signaling induced differentiation with decreased tumorigenic and stem-like cell potential (Stockhausen et al., 2014).

## Perspectives and impact

In summary, therapy development for neurological diseases might be successful for PD with stem cell therapy (Piao et al., 2021), perhaps with a combined approach where growth promoting factors are used to enhance the performance of midbrain dopaminergic neuron grafts (Bjorklund and Parmar, 2021). However, more than 50 years of AD research failed to deliver new therapeutic approaches (Yiannopoulou et al., 2019). Despite the complexity of AD, a recent progress in the genetic analysis of AD patients reviewed here might open up new vistas for research and therapy based on TNFα/NF-κB pathway and neuroprotection. We conclude that TNFα-mediated NF-κB activation essential for neuroprotection is hampered by germ line mutations in many AD patients. This evidence provides a testable hypothesis for reduced NF-κB activation in neurons of AD patients. Mutations may act alone as shown for Sharpin in combinations.

## Author contributions

BK and CK: conceptualization and funding acquisition. BK, LH, JG, and CK: writing–original draft preparation and writing–review and editing. LH and JG: visualization. All authors read and agreed to the published version of the manuscript.
